# Autosomal dominant Marfan syndrome caused by a previously reported recessive *FBN1* variant

**DOI:** 10.1002/mgg3.518

**Published:** 2018-11-28

**Authors:** Eline Overwater, Rifka Efrat, Daniela Q. C. M. Barge‐Schaapveld, Phillis Lakeman, Marjan M. Weiss, Alessandra Maugeri, J. Peter van Tintelen, Arjan C. Houweling

**Affiliations:** ^1^ Department of Clinical Genetics, Amsterdam UMC University of Amsterdam Amsterdam The Netherlands; ^2^ Department of Clinical Genetics Amsterdam UMC, Vrije Universiteit Amsterdam Amsterdam The Netherlands; ^3^ Department of Clinical Genetics Leiden University Medical Center Leiden The Netherlands; ^4^ Department of Genetics University Medical Center Utrecht Utrecht The Netherlands

**Keywords:** abdominal aortic aneurysm, autosomal dominant inheritance, autosomal recessive inheritance, clinical heterogeneity, *FBN1*, Marfan syndrome

## Abstract

**Background:**

Pathogenic variants in *FBN1* cause autosomal dominant Marfan syndrome but can also be found in patients presenting with apparently isolated features of Marfan syndrome. Moreover, several families with autosomal recessive Marfan syndrome caused by pathogenic variants in *FBN1* have been described. The aim of this report was to underline the clinical variability that can be associated with the pathogenic variant c.1453C>T, p.(Arg485Cys) in *FBN1*.

**Methods:**

We provide the clinical details of two autosomal dominant families with this specific *FBN1* variant, which was previously associated with autosomal recessive Marfan syndrome.

**Results:**

Clinical data of 14 individuals carrying this variant from these two families were collected retrospectively. In both families, the diagnosis of autosomal dominant Marfan syndrome was established based on the characteristics of the variant and the phenotype which includes aortic aneurysms and dissections. Of interest, in one of the families, multiple relatives were diagnosed with early onset abdominal aortic aneurysms.

**Conclusion:**

In conclusion, *FBN1* variant c.1453C>T, p.(Arg485Cys) is a pathogenic variant that can cause autosomal dominant Marfan syndrome characterized by a high degree of clinical variability and apparently isolated early onset familial abdominal aortic aneurysms.

## 1 INTRODUCTION

Marfan syndrome (MFS, OMIM #154700) is a multisystem disorder with an estimated prevalence of 1 in 5,000–10,000. MFS is caused by pathogenic variants in *FBN1* (OMIM #134797), encoding fibrillin‐1 and is classically characterized by autosomal dominant inheritance (Dietz et al., 1991). However, several MFS families with an apparently autosomal recessive mode of inheritance have been reported (Fried & Krakowsky, 1977; Hilhorst‐Hofstee et al., 2010; Khan, Bolz, & Bergmann, 2014; Vries, Pals, Odink, & Hamel, 2007). A large proportion of pathogenic *FBN1* variants causing MFS are missense variants, commonly occurring in EGF‐like domains and involving cysteine residue substitutions with a predicted dominant negative effect (Dietz et al., 1993). MFS is classically characterized by skeletal features, ectopia lentis (EL) and thoracic aortic aneurysms and dissections. The diagnosis is based on the revised Ghent criteria (Loeys et al., 2010). Diagnosing MFS is essential since cardiological surveillance and, when indicated, timely aortic surgery is lifesaving (Cameron et al., 2009). The most feared complication of MFS, aortic dissection, is reported in up to 50% of undiagnosed MFS patients and may be the presenting feature of unrecognized MFS (Ammash, Sundt, & Connolly, 2008). Aortic aneurysms and dissections in MFS are typically located in the aortic root and ascending aorta; however, the descending and abdominal aorta may be involved as well (Engelfriet, Boersma, Tijssen, Bouma, & Mulder, 2006; Loeys et al., 2010; Mariucci et al., 2013; Wolfgarten, Krüger, & Gawenda, 2001). Pathogenic variants in *FBN1* may result in classical MFS but have also been reported in families presenting with, for example, apparent isolated thoracic aortic aneurysms and dissections (Wang et al., 2013).

The clinical features of two families with autosomal dominant MFS caused by *FBN1 *variant c.1453C>T, p.(Arg485Cys) and a high rate of abdominal aneurysms is presented here. Homozygosity for this variant was previously reported to cause autosomal recessive MFS in a consanguineous family (Vries et al., 2007). In addition, this variant was reported in a heterozygous state in one patient in a Taiwanese MFS cohort (Hung et al., 2009). Only limited clinical information was provided in this publication. Our report illustrates the importance of clinical follow‐up in *FBN1* mutation carriers, irrespective of previously reported phenotypes associated with that specific variant and suggested mode of inheritance.

## 2 MATERIAL AND METHODS

We retrospectively collected the clinical data of two families (*n* = 14 patients) with the heterozygous c.1453C>T variant in *FBN1 *(NC_000015.9(NM_000138.4):c.1453C>T p.(Arg485Cys)). The families were referred for DNA diagnostics by their clinical geneticists from VU University Medical Center and Leiden University Medical Center, the Netherlands. Informed consent for DNA diagnostics was obtained from all patients. Next‐generation sequencing (NGS) gene panel diagnostics including 13 genes associated with hereditary thoracic aortic disease (*ACTA2*, *COL3A1*, *FBN1*, *FBN2*, *MYH11*, *MYLK*, *PLOD1*, *SLC2A10*, *SMAD3*, *TGFBR1*, *TGFBR2*, *EFEMP2* and *ELN*) was performed. Assessment of the study protocol by our ethics committee was not required since only anonymized data collected during regular patient care were used. Both pedigrees have been slightly adapted in order to ensure privacy.

## 3 RESULTS

**Figure 1:**
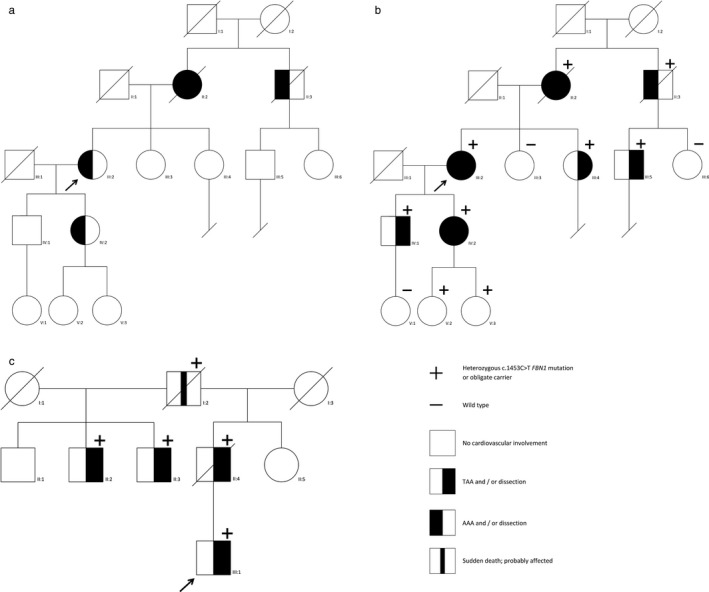
Pedigrees of families 1 and 2. (a) indicates the pedigree at initial presentation of family 1, (b) indicates the pedigree of this family after several years follow‐up. (c) Shows family 2. The proband is indicated with an arrow

Family 1 (Figure 1a indicates the pedigree at initial presentation of the family, Figure 1b indicates the pedigree after several years follow‐up, Table 1.): The proband (III:2) and her daughter (IV:2) were referred for genetic analysis because of the familial occurrence of abdominal aortic aneurysms (AAA) and a type B aortic dissection at older age. Both were diagnosed with an AAA (4.0 cm at the age of 62 years and 5.0 cm at the age of 38 years, respectively). Ophthalmological and physical examination did not reveal any signs of MFS. NGS gene panel diagnostics in IV:2 revealed *FBN1* variant c.1453C>T, p.(Arg485Cys) which was confirmed by Sanger sequencing in III:2. This variant substitutes an arginine by a cysteine in a calcium‐binding(cb)‐EGF‐like domain of fibrillin 1. Introduction of a cysteine in a cb‐EGF‐like domain likely affects the formation of disulfide bridges within the domain. This type of alteration is generally considered to be pathogenic (Loeys et al., 2010). However, because of the nonspecific phenotype and the fact that this variant had been reported in a family with autosomal recessive MFS (Vries et al., 2007), the heterozygous variant was initially classified as likely pathogenic. In order to clarify the clinical significance of this variant in heterozygote state, we offered combined clinical and genetic screening to first‐degree relatives of family members with an aneurysm or dissection. During follow‐up, the proband, her daughter, and several other family members carrying the *FBN1* variant (III:2, III:4, III:5, IV:1, IV:2) were diagnosed with hallmark cardiovascular features of MFS (Figure 1b, Table 1). Of note, no relatives were diagnosed with significant ocular and/or skeletal involvement. Based on the cosegregation and the associated cardiovascular phenotype during follow‐up, the variant was re‐classified to a dominant pathogenic variant and the diagnosis of MFS was established in this family.

**Table 1:** Clinical details of families 1 and 2, and the previously published family (De Vries *et al*). Given the initial uncertainty about the pathogenicity of the variant, cardiologic and/or ophthalmologic evaluation was also performed in several individuals without the variant in family 1 (patient III:3, III:6 and V:1)

Patient	Genotype	Phenotype		
	c.1453C>T^a^	Cardiovascular involvement	Ocular involvement	Skeletal involvement, other features
Family 1				
II:2	OC	Type B dissection 63y, rupture AAA 73y	Unknown	Unknown
II:3	OC	Rupture AAA 80y	Unknown	Unknown
III:2	Het	AAA 62y (E.S.), bilateral subclavian aneurysm 66y (E.S.), TAA 69y	None	Elongated facies, malar hypoplasia
III:4	Het	Type A dissection 59y	None	Malar hypoplasia, pectus carinatum, scoliosis
III:5	Het	Type B dissection 58y	None	None
IV:1	Het	TAA 46y (E.S.)	None	Pectus excavatum, pes plani
IV:2	Het	AAA 38y (E.S.), type B dissection 41y	None	None
V:2	Het	None 18y	None	None
V:3	Het	None 14y	None	None
III:3	WT	None 62y	None	None
III:6	WT	None 48y	None	None
V:1	WT	Unknown	NP	Unknown
Family 2				
I:2	OC	Sudden death 57y	Unknown	Unknown
II:2	Het	Borderline TAA 51y	Myopia>3 dpt	Span to height ratio >1.05
II:3	Het	TAA 47y	None	Downslanting palpebral fissures, elbow contractures, pectus carinatum, pes plani
II:4	OC	Type A dissection 42y, died at 59y heart failure	Unknown	Unknown
III:1	Het	Type A dissection 39y, dilatation coronary artery 39y	NP	Downslanting palpebral fissures, scoliosis, pes plani
De Vries *et al.*				
II:1	Het	None 43y	None	Span to height ratio >1.05, high palate
II:2	Het	None 43y	None	None
II:3	Het	None 37y	None	Span to height ratio >1.05, high palate
II:4	Het	Aortic root 40 mm^b^ 40y	None	None
III:1	Hom	MVP 13y, distal TAA dissection 20y, TAA 22y (E.S.), died 23y	Bilateral lens subluxation, ptosis	Scoliosis, elbow contractures, pectus excavatum, highly arched palate, facial appearance, pneumothorax
III:4	Hom	None 13y	Bilateral lens subluxation, flat cornea	Highly arched palate, lumbosacral dural ectasia

AAA: abdominal aortic aneurysm; E.S.: elective surgery; Het: heterozygous; Hom: homozygous; MVP: mitral valve prolapse; NP: opthalmological examination not performed; OC: obligate carrier; TAA: thoracic aortic aneurysm; WT: wild type; y: age in years.

a Nomenclature *FBN1* variant according to HGVS: NC_000015.9(NM_000138.4):c.1453C>T p.(Arg485Cys).

b Considered normal for BSA.

Family 2 (Figure 1c, Table 1): The proband (III:1) was referred to a clinical genetics outpatient clinic at 39 years of age for genetic counseling after a type A aortic dissection and an aneurysm of a coronary artery. Physical examination revealed downslanting palpebral fissures, scoliosis, and pes plani. His father (II:4) was diagnosed with a type A dissection at the age of 42 years. He died at the age of 59 years due to heart failure. The paternal grandfather (I:2) died suddenly at the age of 57 years. The c.1453C>T, p.(Arg485Cys) variant in *FBN1* was identified by NGS gene panel diagnostics resulting in the diagnosis MFS. Both the father and the paternal grandfather were obligate carriers, since the paternal half‐brothers (II:2 and II:3) of the father were also found to carry the *FBN1* variant. II:2 had an aortic sinus of 4.0 cm and an elongated sinotubular junction at the age of 51 years, whereas II:3 was diagnosed with a thoracic aortic aneurysm of 4.1 cm at the age of 47 years. In addition, both of them had minor signs of MFS at physical and/or ophthalmological examination.

In both families, NGS analysis revealed no other (likely) pathogenic variants or variants of unknown significance.

## 4 DISCUSSION

In total, we present the phenotypic features of 10 genetically confirmed carriers and four obligate carriers of variant c.1453C>T, p.(Arg485Cys) in *FBN1*. These data show that this variant—contrary to earlier observations—is a cause of autosomal dominant MFS. In 2007, de Vries *et al*. reported two cousins with MFS caused by the homozygous c.1453C>T *FBN1* variant, while the four heterozygous parents (ages 37–43 years) did not fulfill the original Ghent criteria for MFS at that time (Loeys et al., 2010). This variant has not been identified in large population databases (ExAC, gnomAD, and GoNL) and has, to our knowledge, only been published in one additional patient from a Taiwanese MFS cohort (Hung et al., 2009).

Though MFS is generally characterized by a dominant mode of inheritance, several other MFS families with an apparently autosomal recessive mode of inheritance have been reported in the literature (Fried & Krakowsky, 1977; Hilhorst‐Hofstee et al., 2010; Khan et al., 2014). Prior to the availability of *FBN1* analysis, Fried and Krakowsky (1977) already suggested the possibility of an autosomal recessive mode of inheritance in MFS. Hilhorst‐Hofstee et al. (2010) described three MFS patients homozygous for *FBN1* variant c.7454A>T, p.(Asp2485Val). In this family, 13 heterozygous relatives were identified, of which only one was diagnosed with MFS based on the original Ghent criteria (Loeys et al., 2010). Khan et al. (2014) reported a 3‐year‐old girl with bilateral lens subluxation and facial features suggestive of MFS carrying *FBN1 *variant c.7258A>C, p.(Asn2420His) homozygously. Her heterozygous parents were unaffected. In addition, several families with autosomal dominant MFS have been described in which family members carrying either homozygous or compound heterozygous *FBN1* variants were more severely affected; however, this was not always the case (Arnaud et al., 2017; Hogue et al., 2013; Karttunen, Raghunath, Lönnqvist, & Peltonen, 1994; VanDijk et al., 2009). Because the c.1453C>T, p.(Arg485Cys) *FBN1* families we describe show a clear autosomal dominant pattern of inheritance, the former report of apparently autosomal recessive MFS due to homozygosity of this variant might be due to age‐dependent penetrance and clinical variability. The age at evaluation of the heterozygous parents of the apparently autosomal recessive family varied between 37 and 43 years, and unfortunately, cardiological follow‐up data are not available. The age at diagnosis of aortic aneurysms and/or dissections in the two presented autosomal dominant families ranged from 38 to 80 years. Therefore, the cardiological phenotype in the unaffected carriers of the variant might still develop during further follow‐up. In the literature, a high degree of clinical variability has been reported concerning the age of onset, the severity, and extent of the clinical manifestations. Different genetic mechanisms, including a second pathogenic variant in another gene associated with thoracic aortic aneurysm and a polygenic model involving multiple modifier loci, are suggested to be a cause of this clinical variability in MFS by recent research (Aubart et al., 2018).

The variability of cardiovascular involvement is also illustrated by family 1 in which the apparent early onset familial AAA was the reason for referral. AAA have been reported as a feature in MFS, and in rare cases even as the presenting feature (Ooijen, 1988; Takayama, Miyata, & Nagawa, 2009; Ugwu et al., 2003; Wolfgarten et al., 2001). Family 2 in this report underlines the importance of DNA testing in individuals with a family history of young patients with AAA and the importance of regular imaging of the abdominal aorta in individuals with Marfan syndrome.

## 5 CONCLUSIONS

This study corroborates the high degree of clinical variability associated with variants in *FBN1 *and provides novel insights into the pattern of inheritance of *FBN1* variant c.1453C>T, p.(Arg485Cys). Moreover, it underlines the importance of clinical follow‐up in heterozygous *FBN1* mutation carriers irrespective of the previously suggested mode of inheritance related to a specific variant.

## CONFLICT OF INTEREST

The authors declare that they have no conflict of interest.
